# Assessment of Finger Fat Pad Effect on CSRR-Based Sensor Scattering Parameters for Non-Invasive Blood Glucose Level Detection

**DOI:** 10.3390/s23010473

**Published:** 2023-01-02

**Authors:** Chaouki Hannachi, Frédérique Deshours, George Alquie, Hamid Kokabi

**Affiliations:** 1 Institut Matériaux Microélectronique Nanosciences de Provence (IM2NP), UMR CNRS 7334, Aix-Marseille Université, 5 Rue Enrico Fermi, 13453 Marseille, France; 2 Laboratoire Génie Électrique et Électronique de Paris (GeePs), UMR 8507 CNRS, Sorbonne Université, 4 Place Jussieu, 75005 Paris, France

**Keywords:** blood, CSRR, Cole–Cole, error, finger fat-pad, glucose, microwave sensors, glucose, scattering parameters

## Abstract

This paper examines the effect of finger fat pad thickness on the accuracy performance of complementary split-ring resonator (CSRR)-based microwave sensors for non-invasive blood glucose level detection. For this purpose, a simplified four-layer Cole–Cole model along with a CSRR-based microwave sensor have been comprehensively analyzed and validated through experimentation. Computed scattering parameter (S-parameter) responses to different fat layer thicknesses are employed to verify the concordance of the studied model with the measurement results. In this respect, a figure of merit (FM) based on the normalized squared difference is introduced to assess the accuracy of the considered Cole–Cole model. We have demonstrated that the analyzed model agrees closely with the experimental validation. In fact, the maximum error difference for all five fingertips does not exceed 1.73 dB over the entire frequency range of interest, from 1 GHz to 4 GHz.

## 1. Introduction

Recent years have seen a considerable growth of interest in developing several glucose-monitoring techniques for diabetes diagnosis. One of the most popular devices is a blood glucose meter based upon a minimal-invasive finger stick test [1,2,3,4]. However, the recent trend is shifting towards microwave planar resonant sensors due to their many interesting features, including low profile, reduced sensing area, and ease of integration with the other conventional planar microwave components [5,6,7,8]. In addition, microwave sensors are preferred due to their capability to measure non-invasively biological samples by emitting an electromagnetic wave that passes through the skin and subcutaneous fat to measure blood [9,10,11,12,13,14,15,16,17,18]. This is thanks to a change in the electrical property of the biological tissue, resulting in a shift to the resonant frequency of the microwave sensor.

Complementary split-ring resonator (CSRR)-based sensors are one of the most widely used devices in several potential applications of microwave sensing, including the characterization of electromagnetic properties of materials, characterization of liquids, and, more particularly, biological material testing [19,20,21,22].

The development of high-sensitivity CSRR microwave planar sensors is required for highly accurate permittivity measurements to monitor the small variations among different biological samples. The sensitivity to variations in glucose levels could be enhanced by increasing the dependency of the minimum transmission resonant frequency on the loaded sample. However, the resonant transmission coefficient could be estimated through the CSRR scattering parameters (S-parameters), which are key for extracting the necessary information for blood glucose concentration measurement [17].

In the context of monitoring glucose levels through the CSRR sensor, the scattering parameter responses are mainly affected by the composition of the characterized fingertip tissue. There are, in fact, several factors affecting the development of fingerprints towards a regular shape (e.g., size, the thickness of subcutaneous fat, degree of stoutness, the growth rate of bone, etc.), which may result in a diversity of fingerprints. Overall, it is thought that the dermatoglyphic pattern of a human fingertip is controlled mainly by genetic factors and mixture permittivity [23,24].

Moreover, tissue composition is generally influenced by the way in which the body distributes excess fat. For instance, in overweight and obese people, some extra fat goes right to their fingers, resulting in non-uniform fat accumulation in the fingertips [25]. Additionally, the fingertip fat pad thicknesses may be differentially modified when pressing on the sensitive region of the non-invasive CSRR-based microwave sensors, contrary to minimal-invasive blood glucose meters where blood glucose levels are measured directly. These scenarios led to discrepancies in the measurement of scattering parameters, which significantly affected the accuracy and the permittivity estimation of the CSRR-based microwave sensor.

Most studies in the literature have been focused on the enhancement of CSRR planar sensors’ sensitivity by improving the accuracy at the permittivity level. However, the proposed approaches do not consider the errors occurring over a wide frequency band since the permittivity is only determined at a given resonant frequency [26]. To ensure a comprehensive assessment of errors, an alternative solution consists of using scattering parameters for identifying and quantifying possible errors related to non-uniform biological tissues over a large frequency band.

In this paper, we employ the CSRR-based microwave non-invasive blood glucose sensors’ scattering parameters to investigate the effect of finger fat pad thickness. It focuses particularly on the change of transmission coefficient responses as key terms for estimating the error due to the variation of fat layer thickness in finger tissues. In this regard, a figure of merit (FM) built upon a theoretical four-layer Cole–Cole fingertip model is introduced for effects analysis purposes. It is described as the squared difference between the computed scattering parameter data at different fat thicknesses and the in-vivo measurement-based scattering parameters of a participant’s fingers. This study aims to provide more flexibility for tolerances compensation and accuracy enhancement of CSRR-based non-invasive microwave sensors for blood glucose level detection.

## 2. Fingertip Tissue Model

**Figure 1:**
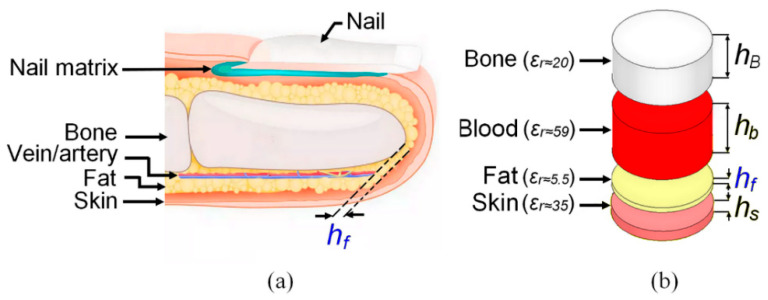
Anatomy of a human fingertip in (**a**) and the adopted Cole–Cole-based four-layers fingertip model in (**b**).

This section deals with the issue of human fingertip electromagnetic modeling. Human tissues are composed of several layers of a complex inhomogeneous mixture of bio-organic materials with different electromagnetic properties, contrary to homogeneous dielectric materials. The anatomy of the human finger is shown in Figure 1a; it typically includes skin, fat, veins/arteries, bone, and nail/nail matrix [27,28].

Voxel-based electromagnetic modeling of human tissues is often employed in the field of computational bio-electromagnetics. Its approach may provide excellent three-dimensional geometric models of the human fingertip. However, that model does not consider the dependency between the complex permittivity of blood and glucose concentration. This dependency is indeed a key factor for non-invasive glucose detection using CSRR sensors. It allows real-time detection of any change in human blood permittivity as a function of glucose concentration. Therefore, an associated Cole–Cole tissue model is introduced to meet analysis requirements since it is widely employed in the electromagnetic modeling of human tissue materials to fit the frequency dependence of the dielectric permittivity [29,30,31,32,33].

According to the Cole–Cole model, the dependency between the complex permittivity ($\hat{\varepsilon }$) of blood and the frequency can be expressed by Equation (1):

(1) $$\hat{\varepsilon }\left(\omega \right)=\varepsilon_{\infty }+\sum_{n}\frac{\Delta \varepsilon_{n}}{1+{\left(j{\omega \tau }_{n}\right)}^{\left(1-\alpha_{n}\right)}}+\frac{\sigma_{i}}{j{\omega \varepsilon }_{0}}$$

where ω is the angular frequency, and *σ*_*i*_ is conductivity.

In order to establish a relationship between blood permittivity and glucose concentration, the blood has been defined as a single pole Cole–Cole material (*n* = 1). Therefore, the corresponding finger tissue model is built using Ansys HFSS 3D electromagnetic simulation software while considering the appropriate parameters of high-frequency permittivity (*ε*_ꝏ_), the magnitude of the dispersion (Δ*ε_n_*), relaxation time constant (*τ_n_*), and dispersion-broadening parameter (*α_n_* = 0.1).

Under the Cole–Cole considerations, the electromagnetic (EM) model of the human fingertip consists of four layers (skin, fat, blood, and bone) having approximately comparable fingertip layer thicknesses of an adult. The thickness of each layer with the associate permittivity (*ε*_r_) is summarized in Table 1 [34], and the considered 3D fingertip electromagnetic model is shown in Figure 1b.

## 3. CSRR-Based Sensor Design and Analysis

**Figure 2:**
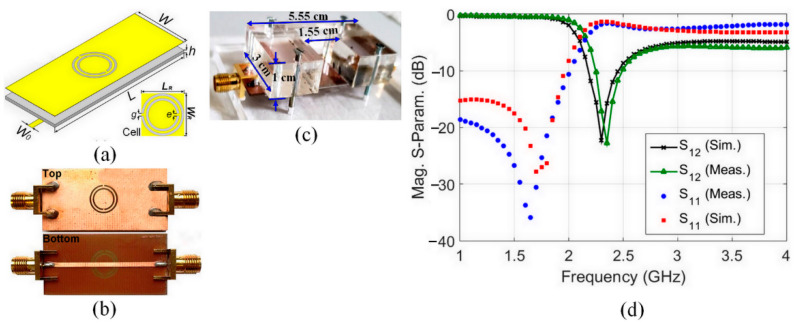
The designed CSRR circuit: (**a**) the geometrical parameters, (**b**) the photograph of the fabricated circuit prototype, (**c**) the geometrical dimensions of the fixture, and (**d**) the measured and simulated S-parameters of the CSRR sensor circuit prior to loading.

The proposed CSRR circuit design is shown in Figure 2a. It was designed to operate at 2.4 GHz on a low-cost FR-4 substrate (*ε*_r_ = 4.4, *tanδ* = 0.025) [35]. The operating frequency of 2.4 GHz was chosen to match the Industrial, Scientific, and Medical (ISM) band 2.4–2.5 GHz for ISM-band biomedical applications. The circuit prototype was fabricated using a Laser-based PCB Prototyping machine (LPKF ProtoLaser S4), as shown in Figure 2b. The overall size of the CSRR circuit is compatible with the fingertip dimension; its geometrical parameters are as follows: *L* = 60, *L_R_* = *W_R_* = 9.079, *W* = 15, *W*_0_ = 1.349, *g* = *e* = 0.500, *h* = 0.730, all units being millimeters. A fixture structure suitable for finger placement was also manufactured to ensure repeatable measurement to incorporate the fabricated CSRR sensor, as shown in Figure 2c. It provides a firm contact of the finger with the CSRR sensing area to perturb the electromagnetic fields and induce noticeable changes in the sensor transmission response.

The fixture design and the selected material were professionally chosen to enable both portability and accuracy throughout the testing process on the sensor. The body of the fixture was made of transparent and rigid plastic material poly (methyl methacrylate) (PMMA). The thickness of the structure used to construct the overall body was 9 mm. The base of the fixture consists of 6 mm PMMA that was cut through an Epilog Fusion M2 40″ 75 W CO_2_ (carbon) laser cutter.

The measured S-parameters of the unloaded CSRR sensor circuit, compared with the simulated one, over the frequency range, from 1 GHz to 4 GHz, are shown in Figure 2d. As can be seen, a very good agreement is achieved, especially across the frequency band of interest (2.4 GHz ISM band). However, between the simulated and measured results, a slight resonant frequency shift, not exceeding 100 MHz (about 4.1% of the resonant frequency), has occurred. This shift in resonant frequency is probably due to the fabrication tolerance and uncertainties in substrate parameters. It should be noted that the accuracy of the proposed finger fat pad thickness effect analysis highly depends on the good correlation between the measured and simulated S-parameters of the unloaded CSRR circuit.

**Figure 3:**
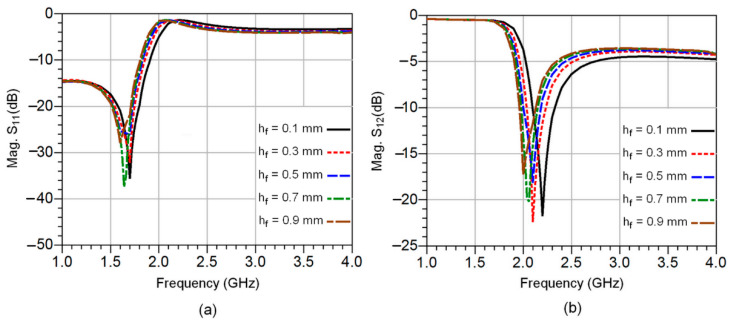
The simulated S-parameters of the CSRR sensor circuit at various fat layer thicknesses: the reflection coefficients in (**a**) and transmission coefficients in (**b**).

The simulated reflection and transmission coefficients for different fat layer thicknesses (*h_f_* = 0.1 mm, *h_f_* = 0.3 mm, *h_f_* = 0.5 mm, *h_f_* = 0.7 mm, and *h_f_* = 0.9 mm) are shown in Figure 3a,b. A change in the resonant frequency as well as in the magnitude of S_21_ (dB) is observed, as shown in Figure 3b. This is due to the variation of the fat layer thickness without considering changes in blood glucose levels.

The resonant frequencies and the associated S_21_ (dB) magnitudes, as well as the computed permittivity, are given in Table 2.

**Figure 4:**
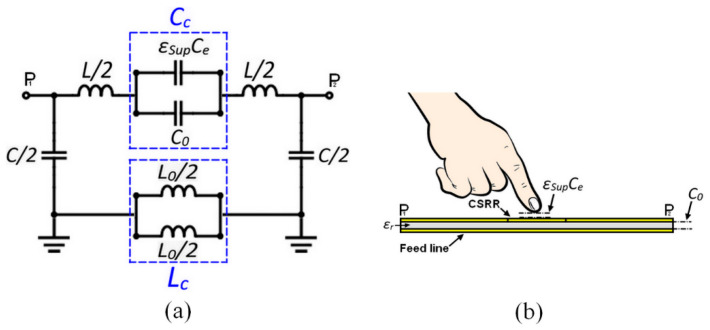
Equivalent circuit model of the proposed sensor in (**a**) and capacitive effects associated with the fingertip placement on the CSRR-sensitive area in (**b**).

The sensor circuit consists of a pair of microstrip transmission lines loaded with a circular CSRR unit cell at the center. The entire CSRR circuit can be modeled by an inductance *L* and capacitance *C* that are related to the microstrip transmission lines. However, the CSRR unit cell could be modeled by a capacitance *C_c_* and the inductance *L_c_*, which are the two key parameters required to determine the sensor’s resonant frequency. The lumped circuit model of the CSRR circuit is presented in Figure 4, and the associated resonant frequency is given by Equation (2):

(2) $$f_{r}=\frac{1}{2\pi \sqrt{L_{c}\cdot C_{c}}}\;$$

It should be noted that the total capacitance *C_c_* is significantly influenced by the electrical characteristics near the CSRR unit cell. By introducing the effects of fingertip loading (termed as superstrate), so the Equation (2) becomes:

(3) $$f_{r}=\frac{1}{\pi \sqrt{L_{0}\cdot \left(C_{0}+\varepsilon_{Sup}C_{e}\right)}}\;$$

where *C*_0_ reports the capacitance between the conductive plates and the circuit dielectric, and *L*_0_ is the inductance of the conductive plates. However, the term *ε_Sup_C_e_* is related to the capacitive effect of the environment due to the superstrate placed on the CSRR sensor.

**Figure 5:**
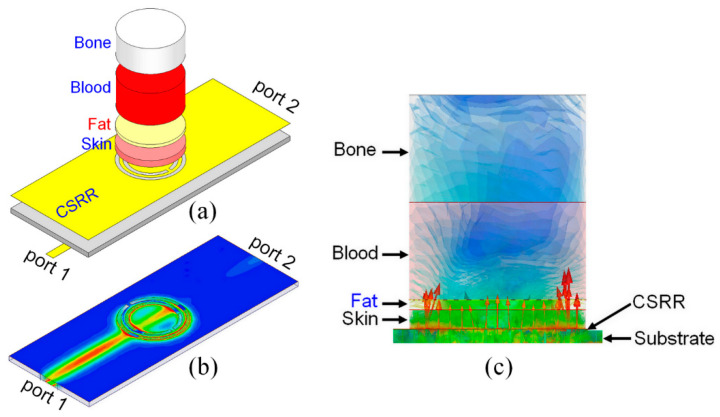
The analyzed four-layers Cole–Cole finger model along with the CSRR sensor using HFSS in (**a**) the electric field distribution on the CSRR at the resonance frequency of 2.3 GHz in (**b**) the distribution of electric vector fields and intensity across the four-layers fingertip model in (**c**).

Figure 5a–c show respectively the analyzed four-layer Cole–Cole model using Ansys HFSS, the electric field distribution on a CSRR, as well as the electric vector fields and intensity distributions across the four-layer fingertip model when loaded on the CSRR sensor at 2.3 GHz. As can be seen, the highest electric field intensity is located at the skin layer and then drastically decreases in the fat layer. The electric vector field becomes maximal at the edge of the CSRR sensor, thus enabling the induction of the blood layer. It is noteworthy that the main consideration in designing the CSRR sensor is to maximize the amount of energy coupled into and back out of tissues. For this purpose, transmission between tissue layers needs to be maximized. Therefore, signal passing between two mediums of different dielectric constant is managed by the transmission coefficient. It is also important to note that the maximum power density occurs at the skin, and the transmitted power decreases nearly exponentially in the skin as a function of depth [36].

## 4. Experimental Analysis of Fat Pad Thickness Effect

The systematic differences in fat pad size between the fingers lead to discrepancies in the scattering parameter measurements, which severely affects the dielectric constant estimation and the accuracy of the CSRR sensor. In this section, the effects of fat layer thickness on the S-parameter responses are analyzed using the four-layer fingertip model in Figure 1b above. In this respect, measured S-parameter data and several simulated sets of S-parameters at different fat pad thicknesses have been extracted from HFSS so that they are treated through Keysight’s Advanced Design System (ADS) software 2020.1.1.

**Figure 6:**
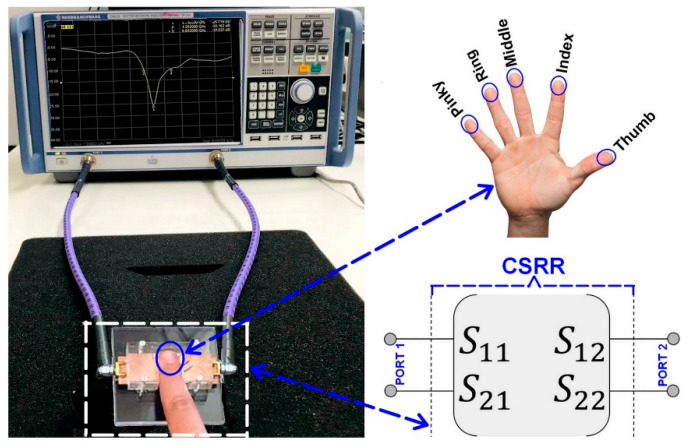
Setup for S-parameter measurement of the manufactured CSRR sensor when loaded with the fingertips of a healthy participant in adult age.

Two-port scattering parameter measurements of the CSRR sensor, when loaded with the fingertips of a healthy participant (fasting blood glucose level: 70–99 mg/dL) at an adult age, were performed using a Rohde & Schwarz ZNB20 vector network analyzer (VNA), as shown in Figure 6. For this purpose, a pair of 50 Ω SMA coaxial connectors were soldered to both ends of the microstrip feed line to enable the measurements when connecting to the VNA. It should be noted that to remove any undesired effects on the measurements and achieve accurate producible measurements, a full two-port calibration was performed using the standard Open-Short-Load technique. The S-parameters data were recorded at an ambient temperature of +25 °C, IF BW (intermediate frequency bandwidth) at 50 Hz, and −10 dB input power for the testing port. The in vivo measurements have been performed by placing each fingertip over the whole sensing region of the sensor and then collecting the S-Parameter results from the vector network analyzer (VNA). The extracted results were correlated with the model-based computed data (the Cole–Cole relaxation model).

**Figure 7:**
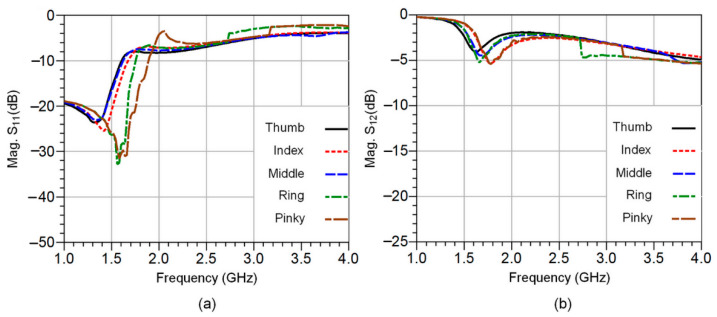
The simulated S-parameters of the CSRR sensor circuit for all five fingertips: the reflection coefficients in (**a**) and transmission coefficients in (**b**).

The measured reflection and transmission coefficients for all five fingertips under similar blood glucose concentration conditions are shown in Figure 7a,b. It should also be noted that during the S-parameter measurement process, a repeatability test is performed over a short period of time and under identical conditions (without changing anything). Moreover, several arrangements have also been taken to ensure improved scattering parameters measurement process.

These include the use of an alcohol wipe before each measurement trial to completely remove dust and humidity on the fingertip and the sensitive area of the CSRR sensor. This leads to resetting the reference resonance at S_21_ before reloading a new fingertip, guaranteeing accurate and repeatable measurements. Thanks to all this, a similar agreement between the results of successive measurements has been achieved.

As can be observed, a change in the resonant frequency as well as in the magnitude of S_21_ (dB) occurred due to the non-uniformity of tissue composition, especially the fat accumulation in the fingertips.

The analysis process consists of using two S-parameter sets having the same number of frequencies and comparing them in order, point by point. The first data set corresponds to the simulated S-parameter of the four-layer fingertip model at different resolutions of the fat layer. However, the second one is the measured S-parameter data from a participant’s fingertips. In both cases, the whole sensing area of the CSRR should be covered in order to minimize modeling errors.

In this perspective, the normalized squared difference between the computed S-parameters data at different fat thicknesses and the measurement-based S-parameters is introduced as an error function (*E_dB_*). It aims to assess the accuracy of the employed model and the impact of a change in fat layer thickness. Its general formula is given by Equation (4):

(4) $$E_{dB}={\left[\frac{\sum_{i=1}^{N}\sum_{j=1}^{N}{\left(\left|S_{ijA}-S_{ijB}\right|\right)}^{2}}{\sum_{i=1}^{N}\sum_{j=1}^{N}{\left(\left|S_{ijB}\right|\right)}^{2}}\right]}_{dB}\;$$

where *S_ijA_* are the computed S-parameters of the four-layer fingertip model, at different fat layer thicknesses (*h_f_* = 0.1 mm, *h_f_* = 0.3 mm, *h_f_* = 0.5 mm, *h_f_* = 0.7 mm, and *h_f_* = 0.9 mm). However, *S_ijB_* is the measured S-parameter data of a participant’s fingertips.

In the case of a reciprocal two-port network, as is the case with the CSRR sensor circuit, Equation (4) becomes:

(5) $$E_{dB}={\left[\frac{{\left|S_{11A}-S_{11B}\right|}^{2}+2{\left|S_{21A}-S_{21B}\right|}^{2}+{\left|S_{22A}-S_{22B}\right|}^{2}}{{\left|S_{11B}\right|}^{2}+2{\left|S_{21B}\right|}^{2}+{\left|S_{22B}\right|}^{2}}\right]}_{dB}\;$$

**Figure 8:**
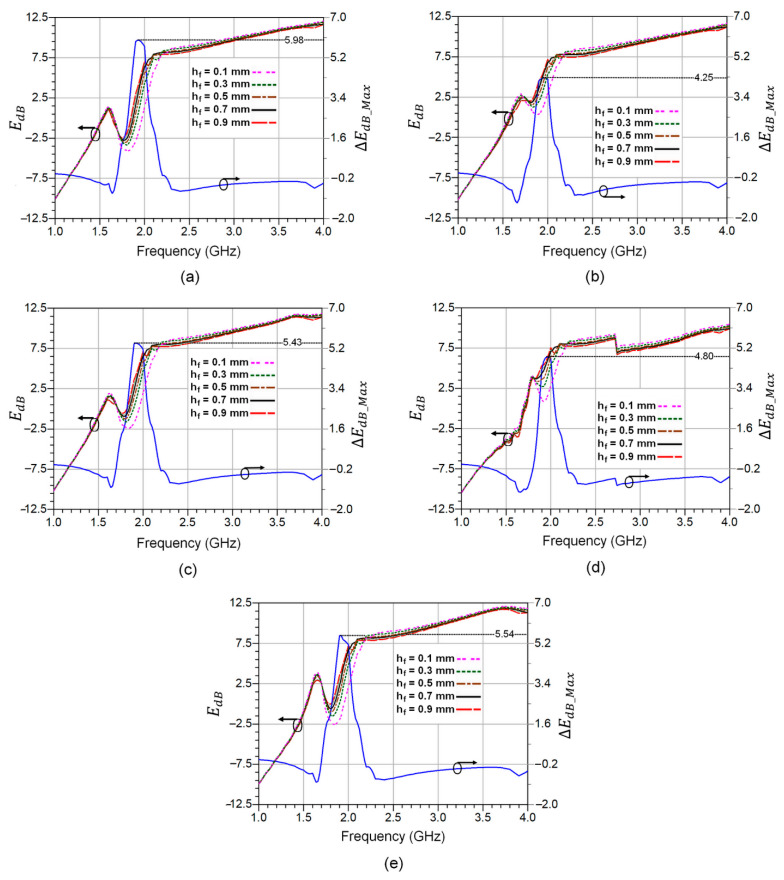
The error *E_dB_* and the maximum error ∆*E_dB_Max_* for all five fingertips at different fat thicknesses as a function of frequency: (**a**) Thumb finger; (**b**) Index finger; (**c**) Middle finger; (**d**) Ring finger; and (**e**) Pinky finger.

The error *E_dB_* as a function of frequency at different fat thicknesses, as well as the maximum error ∆*E_dB_Max_*, is shown in Figure 8a–e, respectively. The range of fat thickness is selected to cover different categories of individuals (lean and obese), which enables determining the maximum error deviation between these two varieties of people. It should be noted that the typical fat thickness value for normal healthy people is 0.5 mm according to the Cole–Cole relaxation model. So, the maximum error resulting from the total variation in fat thickness (∆*h_f_* = 0.8 mm) can be expressed by:

(6) $$\Delta E_{dB\_Max}=E_{dB\left(h=0.9mm\right)}-E_{\left(h=0.1mm\right)}$$

where *E_dB(h = 0.9mm)_* refers to the error at *h_f_* = 0.9 mm and *E_dB(h = 0.1mm)_* is the error at *h_f_* = 0.1 mm.

As can be seen, the change in fat thickness does not significantly affect the scattering parameters in both frequency ranges, from 1 to 1.7 GHz and from 2.3 to 4 GHz. However, the deviation between the errors becomes remarkably high within the 1.7 GHz to 2.3 GHz frequency range.

The maximum error peak achieved is 5.98 dB, corresponding to the thumb finger testing. Whereas, the minimum peak is around 4.25 dB for the index finger. Thus, the error difference for all five fingertips does not exceed 1.73 dB over the whole frequency range of interest, from 1 GHz to 4 GHz. The obtained results show that measurements performed through the index fingertip exhibit low error sensitivity compared to the rest of the fingertips, which confirms the closest agreement with the adopted Cole–Cole-based four-layers model.

## 5. Conclusions

Recent advances in the field of microwave planar sensors have led to a rekindled interest in biological and medical applications enabling real-time and non-invasive measurement of human tissue properties. However, the development of high-sensitivity microwave planar sensors is required for highly accurate complex permittivity measurements to monitor the small variations in various biological tissue samples. In this perspective, uncertainties in the S-parameter measurements of planar CSRR microwave sensors for non-invasive monitoring of blood glucose levels have been investigated. Especially the effect of finger fat pad thickness on scattering parameter responses as a key element in determining dielectric constant for glucose concentrations. The study was carried out based on the computed scattering parameters data of a four-layer finger tissue Cole–Cole model and measured ones from a healthy participant at an adult age. The normalized squared difference was introduced as a figure of merit (FM) for error estimation and accuracy assessment. The achieved results show that the maximum reached additional error is 5.98 dB from the thumb finger, and the minimum additional error is around 4.25 dB from the index finger. However, the maximum error difference for all five fingertips does not exceed 1.73 dB over the entire frequency range of interest, from 1 GHz to 4 GHz. This study provides key inputs for the improvement of the accuracy measurement and error compensation in planar CSRR-based microwave sensors for blood glucose level detection.

## Figures and Tables

**Table 1:** Parameters of the studied Cole–Cole-based four-layers fingertip model.

Parameter	Skin	Fat	Blood	Bone
Thickness	*h_s_* = 1 mm	*h_f_* = 0.5 mm	*h_b_* = 5 mm	*h_B_* = 4 mm
Permittivity	35	5.5	59	20

**Table 2:** Resonant frequencies, S_21_ (dB) magnitudes, and dielectric constants for different fat layer thicknesses.

Fat Thick. h_f_ (mm)	0.1	0.3	0.5	0.7	0.9
Res. Freq. *f_r_* (GHz)	2.2	2.1	2.08	2.05	2
Δ*f*_r_ (GHz)	0.1	0.2	0.22	0.25	0.3
S_12_ (dB)	−21.78	−22.34	−18.16	−22.76	−17.27
Permittivity	4.97	6.47	6.98	7.33	8.83

## Data Availability

Data generated during the study are contained within the article.
